# Genetic variability of transcript abundance in pig peri-mortem skeletal muscle: eQTL localized genes involved in stress response, cell death, muscle disorders and metabolism

**DOI:** 10.1186/1471-2164-12-548

**Published:** 2011-11-04

**Authors:** Laurence Liaubet, Valérie Lobjois, Thomas Faraut, Aurélie Tircazes, Francis Benne, Nathalie Iannuccelli, José Pires, Jérome Glénisson, Annie Robic, Pascale Le Roy, Magali SanCristobal, Pierre Cherel

**Affiliations:** 1 Laboratoire de Génétique Cellulaire, INRA UMR444, Chemin de Borde Rouge, F-31326 Castanet-Tolosan, France; 2 Hendrix Genetics RTC, 100 avenue Denis Papin, F-45808 St Jean en Braye Cedex, France; 3 INRA UMR598 Génétique Animale, F-35042 Rennes, France

**Keywords:** eQTL, muscle, transcriptome, genetical genomics, systems biology, pig

## Abstract

**Background:**

The genetics of transcript-level variation is an exciting field that has recently given rise to many studies. Genetical genomics studies have mainly focused on cell lines, blood cells or adipose tissues, from human clinical samples or mice inbred lines. Few eQTL studies have focused on animal tissues sampled from outbred populations to reflect natural genetic variation of gene expression levels in animals. In this work, we analyzed gene expression in a whole tissue, pig skeletal muscle sampled from individuals from a half sib F2 family shortly after slaughtering.

**Results:**

QTL detection on transcriptome measurements was performed on a family structured population. The analysis identified 335 eQTLs affecting the expression of 272 transcripts. The ontologic annotation of these eQTLs revealed an over-representation of genes encoding proteins involved in processes that are expected to be induced during muscle development and metabolism, cell morphology, assembly and organization and also in stress response and apoptosis. A gene functional network approach was used to evidence existing biological relationships between all the genes whose expression levels are influenced by eQTLs. eQTLs localization revealed a significant clustered organization of about half the genes located on segments of chromosome 1, 2, 10, 13, 16, and 18. Finally, the combined expression and genetic approaches pointed to putative *cis*-drivers of gene expression programs in skeletal muscle as *COQ4 *(SSC1), *LOC100513192 *(SSC18) where both the gene transcription unit and the eQTL affecting its expression level were shown to be localized in the same genomic region. This suggests *cis*-causing genetic polymorphims affecting gene expression levels, with (e.g. *COQ4*) or without (e.g. *LOC100513192*) potential pleiotropic effects that affect the expression of other genes (cluster of *trans*-eQTLs).

**Conclusion:**

Genetic analysis of transcription levels revealed dependence among molecular phenotypes as being affected by variation at the same loci. We observed the genetic variation of molecular phenotypes in a specific situation of cellular stress thus contributing to a better description of muscle physiologic response. In turn, this suggests that large amounts of genetic variation, mediated through transcriptional networks, can drive transient cell response phenotypes and contribute to organismal adaptative potential.

## Background

Most quantitative phenotypes or disease susceptibility factors are complex traits that are the result of interplay between genetic variation and environmental exposure. In animal genetics, many quantitative trait loci (QTL) have been described as components of genetic variation in muscle physiology, disease resistance, and reproduction traits [1]. But positional cloning of polymorphisms underlying those QTLs is a long process, as is understanding the molecular cascades that lead to the phenotypes observed. The identification of expression QTLs (eQTLs) should help to characterize the primary effects of genetic variation and provide opportunities to understand the molecular processes that are affected by this variation. Genes whose transcripts are affected by these eQTLs do not necessarily embed causative polymorphisms, but the distribution of eQTL localizations joined to transcript correlation structure enables functional groups of genes to be defined [2]. Variation in gene expression is thought to be responsible for a large part of the phenotypic variation observed in natural populations. Changes in gene regulation have been found to underlie adaptative phenotypes in different species [3,4]. For all these reasons, eQTL mapping studies is a new powerful tool to identify genetic variants that regulate gene expression [5-9]. Transcriptome analysis using microarrays measures the expression level, the phenotype in eQTL analysis, of many genes, and segregation of genetic markers within families allows mapping of the loci affecting those phenotypes to specific genomic regions. Global eQTL analyses have enabled detection of *cis *genetic variation controlling individual genes and significant clustered *trans *eQTLs that regulate group of genes [10].

Muscle is a highly organized and complex tissue whose properties are likely to be determined at different levels. In farm animals, muscle fiber characteristics play a key role in meat quality. Myofibril type ontogenesis occurs during the embryonic period and continues until the early postnatal period in the largest species (cattle, sheep, and pigs) and the total number of fibers is fixed. Contractile and metabolic differentiations occur soon after birth in pig [11]. In mammals, post-natal muscle tissue constitutes about 50% of the body mass and enables high potential of plasticity in response to metabolic variation (stress, heat production, exercise, injury, nutrient storage and supply, etc.) [12,13]. This plasticity corresponds to the possibility of changes in gene expression in response to rapid environmental events, or with remodeling, the renewal of muscle cells as satellite cells [14]. Consequently, muscle is one of the best examples of a tissue with an inherent adaptation capacity to sustain not only locomotion but also a number of life-sustaining processes [15].

Other authors have explored eQTL mapping in pig [16-19]. Cardoso *et al*. (2008) presented a simulated data to describe a way to design pig eQTL experiment and to select animals for eQTL mapping. Ponsuksili *et al*. (2008, 2010) explored eQTLs associated with water holding capacity, an important meat quality trait [16] and recently reported on a global comparative analysis between eQTLs and several meat quality traits [18]. In a QTL project for expression profiling, Steibel *et al*. (2011) analyzed eQTLs obtained with 176 F2 animals chosen for their extreme phenotypes for either loin muscle area or backfat depth. These authors compared the eQTL and the QTL results to identify candidate genes [19].

The aim of our study was to analyze muscle eQTLs in one F2 family without preselecting trait-associated gene expression levels. Using muscle samples from 57 half sibs, we identified 335 eQTLs that characterize genetic variation in the expression levels of 272 genes. Moreover, the overlapping genomic localization of eQTLs and some transcribed genes suggests candidate genes for embedding *cis*-acting causative polymorphisms. Alternatively, clustered *trans*-eQTLs genomic regions can define sets of genes potentially affected by the same genetic polymorphisms through shared cellular functions.

## Results

### The expression levels of 272 genes are genetically regulated by one to four eQTLs

**Figure 1:**
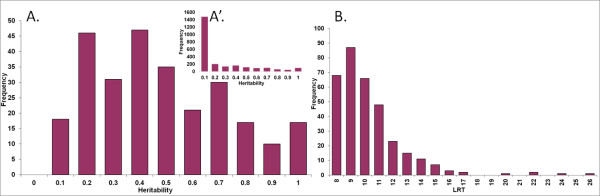
**Distribution of heritability estimates (A) for the 272 genes with eQTLs and (A') for the 2464 genes expressed in muscle and (B) histogram of the LRT values of the 272 genes**. Detailed information is given in Additional files 1 and 3.

Using a cDNA microarray, we measured the expression levels for 2,454 transcripts in 57 *Longissimus lumborum *samples collected on pig carcasses 20 minutes after stuning and exsanguination. Heritability of expression levels was estimated to select transcripts for which the expression level is driven by inherited factors (Figure 1A) and to enable a QTL segregation to be tested against a polygenic additive only model (null hypothesis). Heritability of at least 5% was estimated for 1,057 of these transcripts which were then analyzed for QTL detection. The large proportion of transcripts for which the estimate additive variance was not significant (1,397/2,454: 57%) reflects (i) RNA species with little or no variance, and (ii) the limited power of our design, where, in contrast to paternal haplotype segregation, the estimation of additive variance is only possible for the dam side. The average heritability across the 1,057 transcripts deemed heritable (h^2 ^> 0.05) was of 0.18 (Figure 1A').

Analysing QTLs that affected expression of 1,057 transcripts deemed heritable, we detected 335 eQTLs, significant at a chromosome-wide threshold of 1%, that regulate the expression of 272 genes. Details of eQTL detection including maximum LRT, most likely position on the genetic map and estimated heritability are given for each of the 272 transcripts with identified eQTLs in Additional file 1. Accounting for the large number of tests performed (18 chromosmes, 1,057 traits) using a canonical transformation of traits suggests an overall false discovery rate of 15% (see Methods). The 1% chromosome-wise thresholds for LRT are listed in Additional file 2. The average heritability was of 0.45 ± 0.25 for the 272 transcripts with at least one significant eQTL.

Additional file 3 presents all the 335 LRT values and Additional file 1 summarizes the loci identified as affecting the expression level for the 272 transcripts. LRT values ranged from 7.5 to 25.3 (Figure 1B). The Table 1 and Additional file 1 (the last line of the table) also list the number of genes whose expression was shown to be genetically regulated by loci localized on each chromosome; e.g. the expression of 49 genes was genetically regulated by at least one gene on SSC1.

**Table 1:** Description of the 335 eQTLs localized on swine chromosomes.

	eQTL			
	
	number of eQTL	clustered eQTL	cM	adjusted p-value
**SSC1**	**49**	6	83-101	NS
		**31**	**133-149**	**9.06E-61**

**SSC2**	**28**	**18**	**51-77**	**3.14E-12**

SSC3	16	4	39-55	NS

SSC4	17	4	61-81	NS
		5	100-131	NS
		4	149-151	NS

SSC5	13	4	1-39	NS
		5	100-150	NS

SSC6	13	5	20-60	NS
		5	80-140	NS

SSC7	12	3	60-80	NS
		6	160-end	NS

SSC8	23	5	0-60	NS
		11	63-109	NS
		8	125-180	NS

SSC9	6			

**SSC10**	**29**	**25**	**1-5**	**9.09E-38**

SSC11	3			

SSC12	5			

**SSC13**	**29**	**18**	**1-39**	**1.69E-06**
		5	85-120	NS

SSC14	7	5	40-60	NS

SSC15	6			

**SSC16**	**44**	**36**	**41-65**	**8.87E-68**

SSC17	11	6	0-15	NS

**SSC18**	**24**	8	1-10	NS
		**15**	**20-55**	**0.00010**

Total	335	242		
Significant total		**154**		

72	% of the eQTL are co-localized.			
46	% of the eQTL are co-localized and this enrichment is significant at the chromosome level.			

The number of eQTLs is given for each chromosome. This number is in bold when the chromosome level is significantly high (Bonferroni-corrected p-value). The position of the clusters is given in cM (details are given in Additional files 1, 3 and 4).

**Figure 2:**
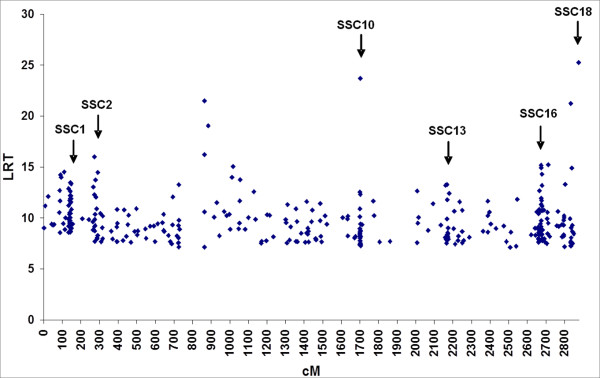
**Localization of the 335 eQTLs along the genome**. Around the half of the eQTLs were organized in clusters on chromosomes 1, 2, 10, 13, 16 and 18 (see Table 1 and Additional files 1 and 3 for details).

Figure 2 shows the locations and LRT values of the 335 identified eQTLs. These eQTLs were not randomly distributed along the genome, 46% of them were grouped in six clusters. The clusters were on SSC1 at 133-149 cM (31 transcripts), on SSC2 at 51-77 cM (18 transcripts), on SSC10 at 1-5 cM (25 transcripts), on SSC13 at 15-29 cM (18 transcripts), on SSC16 at 41-65 cM (36 transcripts), and on SSC18 at 20-55 cM (14 transcripts) (Table 1 and Additional files 1 and 3). Six clusters of eQTL were each associated to a highly significant chisquare test for enrichment of eQTL in the referenced genome segment (p value < 10^-5^, Additional file 4).

The 272 genes were affected by one to four significant eQTLs each: one gene was associated with four significant eQTLs, 11 genes with three significant eQTLs, 38 with two eQTLs, and 222 with only one eQTL.

### Gene mapping and annotation revealed mainly *trans*-eQTL and few *cis*-eQTL

Knowing the chromosomal localization of genes is essential in an eQTL study. Mapping and annotations are described in Methods and results are summarized in Additional files 1 (transcripts) and 2 (genetic markers). Most of the transcripts (213, 78%) were directly localized on the pig genomic sequence (Sscrofa9). For 88% of the transcripts, we obtained an alignment with a BAC sequence, which, excepted for five transcripts, was localized on pig chromosomes allowing 33 more transcripts to be localized on chromosomes. This enabled a total of 246 transcripts to be localized on pig chromosomes (90%).

As a result of gene annotation, 71% of the transcripts were annotated (193/272). Annotation results are given in Additional file 1 and details in Additional file 5. We observed a low redundancy with 186 unique genes out of the 193 genes annotated (less than 4%).

The final important information provided by gene and eQTL mapping was the identification of eQTLs colocalized with gene transcription units, i.e. *cis*-eQTL. Identifying of *cis*-eQTL is not easy, owing to the very large confidence intervals associated with each eQTL. To compare respective eQTL and gene localizations, we had to assign both locations to common reference coordinates. Practically speaking, we used genome assembly as a common reference, having localized the position on the genome assembly (Additionnal file 2) of the STS genetic markers used to build the genetic maps (eQTL position reference). We considered as putative *cis*-eQTLs those where genetic markers flanking the most likely eQTL position on the genetic map also bracketed the gene position on chromosome. This condition applied to a total of 18 eQTLs and affected the trancription of the following genes: *EEF1A1*, *COQ4 *and *CR939593 *on SSC1; *IK *and *PDLIM7 *on SSC2*; MDH2 *on SSC3; *PCBP2*, *HNRNPA1*, *MGP *and *EMG1 *on SSC5; *TMEM201 *on SSC6*; EAPP *on SSC7; *THYN1 *on SSC9; *ALDH2 *and *ACTN2 *on SSC14; *BX676048 *and *OCLN *on SSC16; and *LOC100513192 *on SSC18. The average distance between a gene and the eQTL closest bracketing marker was 15.3 Mb (see Additional file 3 for details). Some of these *cis*-eQTLs were isolated, such as *LOC100513192*, and some others (e.g. *COQ4, PDLIM7) *are co-localized with clusters of *trans*-eQTLs.

### Ontological and functional description of the eQTLs

A functional annotation of the genes identified as being genetically regulated in muscle tissue should provide new insights into the molecular mechanisms that determine the muscle phenotypes. In this study, we did not preselect genes, e.g. genes differentially expressed for one trait. All 272 genes analyzed for function were identified solely on the basis of their expression being affected by one of the eQTLs identified in muscle tissue.

**Figure 3:**
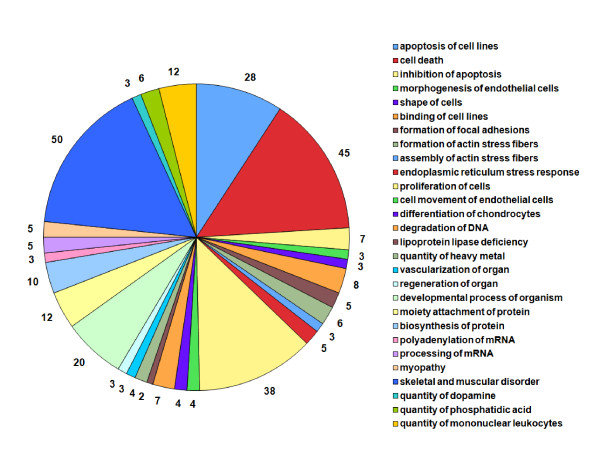
**The 28 function enrichment analysis of genetically regulated genes (IPA)**. The pie chart shows the 28 significant (p-value < 0.01) biological functions for the 186 annotated genes with genetically regulated expression (details in Additional file 7). These functions are organized by category and the number of genes involved is given for each function.

As a consequence of partial gene annotation, only 186 genes were practically used for the functional analysis. Two approaches were used to explore the biological functions regulated by the genes involved in these eQTLs. First, we used a systematic ontologic analysis with the EASE software. Ontologic (biological process, molecular function, and cellular component) annotations were obtained for 123 genes and 33 genes had a KEGG pathway annotation. The systematic results of the gene ontology and the Kegg pathways are summarized in Additional file 6. The 186 genes regulated by at least one eQTL were analyzed using the IPA software (Ingenuity Pathway Analysis). IPA took into account 145 genes for the enrichment analysis of biological functions (ontologies and KEGG pathways, Figure 3 and Additional file 7). Twenty-eight biological functions were identified (pvalue < 0.01). The pie chart in Figure 3, shows the functions organized by category from cell death to biochemistry. The most significant functions concern lipoprotein lipase deficiency just before cell death, cell morphology, stress response, transcription and translation, and other more muscle specific functions.

**Figure 4:**
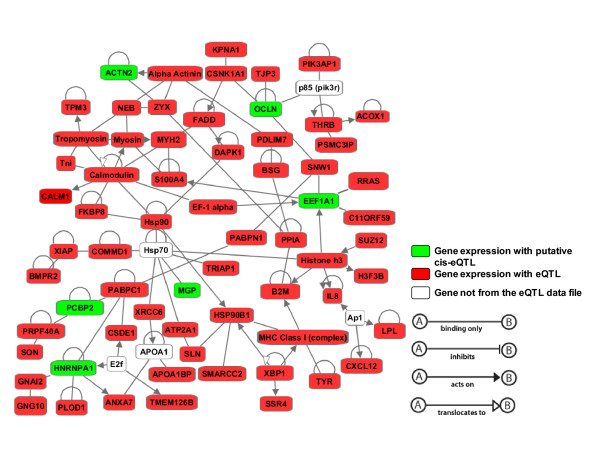
**Network of 57 eQTLs with cellular movement, cell-to-cell signaling and interaction, system development and function as main biological functions**. This network has been constructed with Ingenuity Pathways Analysis and includes 6 putative *cis*-eQTL (green) and 51 *trans*-eQTLs (red).

Among the 186 genes, IPA identified 169 as "network elligible" to propose biological networks based on bibliographic data (Figure 4 and Additional file 8). IPA constructed three highly significant networks. The first network had a score of 81 (each including 57 genes), the second network had a score of 79 (including 55 genes), and the last had a score of 61 (including 47 genes). The three networks are presented in Additional file 8 (as 8.1, 8.2 and 8.3). The top biolological functions associated with the three networks were the muscle development and physiology (networks 1 and 2), cell metabolism (network 3), cellular movement, cell-to-cell signaling and interaction (network 1), and protein synthesis and post-translational modification (networks 2 and 3). These three networks included 80% of the genes identified as network elligible (149/169) by the IPA software, then suggesting that these genes, which where not selected according to a specific trait or physiological function, nevertheless reflected shared biological functions. This might be understood as if the largest components of the genetic regulation of gene expression in this tissue and sampling condition do not necessarly reflect muscle ontology or physiology associated functions but instead are depicting the most regulated cell functions at sampling point, where gene expression regulation appears as a coordinated cell response to environmental challenges. Moreover, a combined large network (Additional file 8, network 8.4) was constructed with the three networks with some genes/eQTLs shared by two networks: *ACOX1*, *CSDE1, OCLN, PABPN1, PIK3AP1 *joined networks 1 and 2, *CSDE1 *joined networks 2 and 3, while *CSDE1, NEB, SMARCC2 *joined networks 1 and 3.

**Figure 5:**
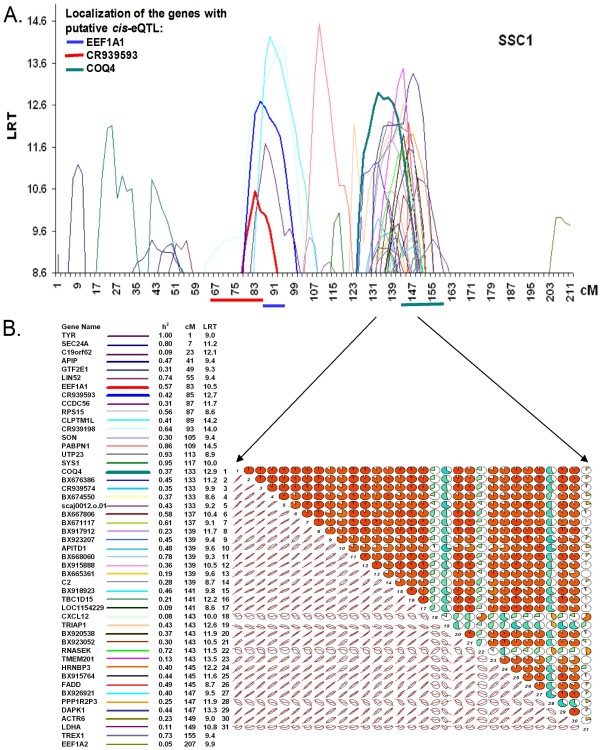
**eQTL mapping on SSC1 (A) and correlogram of the predicted eQTL effects for the genes involved in the 133-149 *trans*-eQTL cluster (B)**. A. The chromosome-wise threshold of 1% (based on simulations) corresponds to the lowest LRT value of 8.6 on the graph. All the 49 eQTL on SSC1 are mapped. The *trans*-eQTLs are represented by narrow lines whereas the three putative *cis*-eQTL are represented by thick lines (*COQ4, EEF1A1 and CR939593*). B. The correlogram comprises the 31 genes with eQTL in the 133-149 cM cluster with the colored lines used for mapping. The correlogram includes a circle and an ellipse. Each circle is shaded red or green depending on the sign (+ and - respectively) of the correlation, and with the intensity of color scaled 0-100% in proportion with the magnitude of the correlation. The ellipse is a schematic scatterplot matrix and each panel depict the patterns of relations among variables with confidence ellipse and smoothed curve.

The low proportion of genes with functional annotation found in clusters prevented us from performing a significant functional annotation within each cluster. For example, one of the largest clusters containing 31 eQTLs on chromosome 1 (Figure 5) included only 14 genes with functional annotation. An alternative indirect way to infer a putative common regulation of genes included in the same cluster of *trans*-eQTL would be to assess the relationships between the estimated eQTLs effects for the different levels of expression involved. This might not include all cases of shared genetic determinism (when the same polymorphism has different effects on gene expression for different genes) but nonetheless might highlight the easiest case of a parallel response to the same polymorphism. Accordingly, we visualized the correlation structure among the individual eQTL effects predicted for these 31 transcript levels (Figure 5A) at a median position (141 cM) and observed that 26 of these transcripts were affected by highly correlated eQTL effects (Figure 5B). The mean of Pearson correlation coefficients among the eQTL effects affecting these 26 transcripts levels was 0.89. The corresponding residuals from the same analyses (i.e. after correction for eQTL effects) showed substantial but lower correlations between the transcripts involved (mean correlation of 0.67). Moreover, the combination of concentration ellipses and loess smoothing (as schematic visual representation) summarizes the linear and possibly nonlinear association underlying the complexity of molecular regulation we were searching for [20].

**Figure 6:**
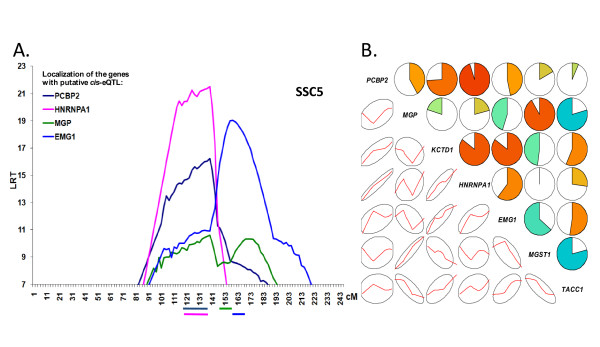
**Four putative *cis*-eQTLs mapped on SSC5 (A) and correlogram of the predicted eQTL effects for the seven gene with an eQTL on 139-205 cM on SSC5 (B)**. A. The chromosome-wise threshold of 1% (based on simulations) corresponds to the lowest LRT value of 7.5 on the graph. Only the four putative *cis*-eQTL are presented. B. The correlogram is for the seven genes with an eQTL in the 139-205 cM (maximum LRT values for each gene on the chromosome 5) with the color lines used for mapping.

Within the 129-205 cM interval on chromosome 5 where four putative *cis*-eQTLs co-localized with a smaller group of *trans*-eQTLs, the correlogram of predicted eQTL effects at the 141 cM mid-cluster position revealed two functional groups of transcripts, where correlated eQTL effects are affected the expression of *PCBP2*, *HNRNPA1, KCTD1*, and *EMG1*, while *MGP*, *MGST1*, and *TACC1 *are affected by a distantly related set of correlated eQTL effects (Figure 6). The mean of absolute values of Pearson correlation coefficients among *PCBP2*, *HNRNPA1, KCTD1*, and *EMG1 *eQTL effects was 0.81 (the mean correlation of corresponding residuals was 0.41). The mean of absolute value of Pearson correlation coefficients among *MGP*, *MGST1*, and *TACC1 *eQTL effects was 0.78 (the mean correlation of the corresponding residuals was 0.43).

## Discussion

### A total of 272 genes are genetically regulated in muscle tissue with an average heritability of 0.45 in a F2 pig half-sib family

The specific interest of this work was first focusing on global gene expression regulation without prior selection of transcripts for association with a particular trait, and second using a family structured population that enabled genetic analysis. We observed differences in heritability estimates between the group of 1,057 transcripts subjected to QTL analysis (average h^2 ^0.18) and trancripts affected by the eQTLs identified (h² 0.45). This is consistent with the expectation that significant QTL contribute to polygenic additive variation estimates even when this is not the best possible model. The size of our dataset limited the power of detection and it is likely that we were not able to detect all the loci involved in the regulation of gene expression. In a review of genetical genomics studies done with SNP and genome wide associations, Cookson *et al*. (2009) observed that the QTL underlying genetic variability of expression is often detected for only less than 20% of the estimated trait heritability [21]. Cookson *et al*. explained that some genetic or other factors like family clustering, sample stratification and genetic markers informativity are limiting the power to detect other loci. Nevertheless, the starting point is that variation of expression is not only a result of transcriptional regulation but also of genetic regulation in order to explain part of the phenotypic variability in a constant environment.

Another original choice was using tissue RNA sampled in individuals from an outbred population (but in a controlled environment) to analyze genetic regulation of gene expression. Most published eQTL studies used inbred lines of model organisms and Gilad *et al*. (2008) [10] suggested that it would be more informative to evaluate regulation of expression in populations carrying natural sources of genetic variation. Example of such representative genetic variability could be found in human populations, outbred mice [22,23] or, to a lesser extent, pig lines (from breeding programs). In our work, it is worth noting that this F2 family was constructed from two synthetic lines (a synthetic line and an outbred line) intercrossed for two generations in order to increase the segregation of genetic variability in F2 animals. All animals analyzed were raised in the same conditions, allowing us to control the effect of the environment, while exploring a physiological context (post slaughtering/hot ischemia) which is very specific of meat harvesting in livestock animals raised for meat but which could be also considered as an animal model of ischemia in skeletal muscle. In human, the first eQTL studies orginally focused on transformed lymphocyte cell lines (mainly the HapMap lymphoblastoid cell lines). New studies have been undertaken on clinical samples of liver, adipose tissue, postmortem brain [24-28], although sample access and standardization remains limiting. Animal studies in outbred populations offer an attractive combination of standardized environment, large family sizes allowing linkage studies and more easily accessible tissues.

### The 335 eQTLs are mainly *trans*-eQTLs revealing genes with shared biological functions

In our study, we based the precise localization of the transcribed genes on highly reliable sequence homologies of cDNA used as probes on the genome sequence (related to a genome sequence in progress, version 9 available). It is also worth noting that the method we used to quantify gene expression, which is based on cDNA microarray hybridization, is relatively immune to artefactual *cis*-eQTL identification [29-31] where DNA polymorphisms such as SNPs can modify the hybrization efficiency of oligo probes and lead to an apparent change in gene expression, in fact contributed by these loci. In these conditions, we identified a small proportion of putative *cis*-eQTL (18 putative *cis*-eQTLs localized in 335 eQTLs) like in a recent comparable study on pig muscle reported by Ponsuksili *et al*. (2010) where 35 putative *cis*-eQTLs were identified [18]. An observation common to our and other eQTL studies is that the significance of detection is generally higher for *cis*-eQTL than for other eQTLs. In our study, the highest LRT value was found for a putative *cis*-eQTL *LOC100513192 *on SSC18. This was expected as *cis*-eQTLs are supposed to transfer the contribution of a given genomic polymorphism directly to local gene expression, and not indirectly via actions that occur along pathways or via gene interactions, all of which can create background noise thus hindering the detection of QTL. This was also the case in eQTL studies in mouse, rat and human [10,32] using very large oligo arrays for gene expression measurements and large sets of SNPs genetic markers in which multitesting to avoid false discovery mainly highlighted highly significant *cis*-eQTLs and few *trans*-eQTLs. This is a powerful way to directly identify SNP polymorphisms as putative regulators of local gene expression but it does not provide information on the architecture of gene and molecular pathway regulation.

In genetic analysis of physiology and pig meat quality traits, many QTLs have been reported but few led to the identification of genes and causal polymorphisms mainly because of the complexity of trait determinism and epistatic regulations [16,33,34]. This is why we hoped that eQTL studies would help to decipher how this complexity is established. In this context, it was interesting to identify eQTL for genes without prior selection and to identify only six clusters of *trans*-eQTLs suggesting a shared genetic regulation (for about half the genes), but also that the expression levels of some genes are regulated by more than one eQTL.

Constructing bibliographic gene networks (using Ingenuity software), we obtained three highly significant networks for 149 genes (out of the total of 186 annotated genes) suggesting that the genes identified for their genetic regulation mostly work together (Figure 4). Moreover the three networks can be merged into a single network underlying the shared biological functions regulated by these eQTLs/genes (Network 8.4 in Additional file 8). Networks 1 and 2 mainly underlie the development and organization of muscle tissue whereas network 3 is involved in cell metabolism. We were not able to allocate a specific cluster of *trans*-eQTLs to one network or vice versa to be able to say that one network is specifically regulated by one or more clusters of eQTLs. Likewise, as discussed below in the case of the cluster of eQTLs on chromosome 1 (and in Results), the low number of annotated genes per cluster did not allow us to really perform functional analysis of each cluster.

However, we performed enrichment analysis of the biological functions regulated by IPA (Figure 3). Among the top biological functions identified, the cell cycle and death (tumorigenesis, proliferation, apoptosis, cell death) were the major functions identified. Forty-seven genes (28% of the genes named elligible by IPA) are involved in cell death (including apoptosis and inhibition of apoptosis). This may be related to the way muscle tissues were sampled (at slaughtering) and may be correlated with the effect of stress, which was the second top function identified. These functions were previously considered as related to meat quality traits [34] and stress is well known to be responsible for meat defects [35-38]. Some biological functions are related to the organization of muscle cells and tissue including connective tissue, focal adhesion, filaments, and vascularization. Others are more related to muscle signal transduction pathways like the quantity of heavy metals, phosphatidic acid and calcium flux. Fundamental molecular processes such as degradation of DNA, mRNA processing, protein biosynthesis and modification are also involved.

### The genetic regulation of muscle gene expression by the 133-149 cM locus on chromosome 1 is involved in stress response

To explain the genetic regulation of a cluster of eQTLs, we use the example of one of the largest cluster in our study. It concerns chromosome 1 at locus 133-149 cM which regulates the expression of 31 genes (Figure 5). The bibliographic network of this cluster involved nine genes that shared the biological function cell death (data not shown), obtained with Ingenuity software, including *COQ4 *a putative *cis*-eQTL co-localized with the cluster. We explored the correlation structure of gene expression of members of this eQTL cluster to identify possible coregulations and to distinguish between groups of genes affected (or not) by the same genetic variation (individual predicted eQTL effect, Figure 5B). This observation suggests a shared genetic determinant affecting 26 transcripts levels out of the 31, while above and beyond eQTL effects, the overall phenotypic correlation would be consistent with co-regulation of these transcripts in the same pathway or transcriptional network. This would imply parallel responses not only to an eQTL allele, but also to all alternative genetic and environmental effectors that affect this expression network. This analysis also highlighted functional heterogenity within a group of co-localized eQTLs, where colocalization can result not only from multiple transcripts affected by the same locus, but also from independent genetic effets localized on the same chromosome segment.

Nevertheless, we observed that expression of most of the genes in the cluster was highly correlated among genes and particulary with *COQ4*. In this context, the putative *cis*-eQTL *COQ4*, can be proposed as a candidate primary effector of an identified eQTL locus, affecting the whole group of transcripts downstream. This was not originally expected firstly because of the co-localization of eQTLs within a cluster could be random, and secondly because if gene expression is influenced by shared genetic regulation, this is far from implying a tight coregulation owing to alternative sources of regulations from the environment or from other loci for each gene. Gene *COQ4 *is located in the same genetic interval (*cis*-eQTL) and has recently been identified in human [39] as coding for one of the Coenzyme Q (CoQ) proteins, which are small lipophilic molecules that transport electrons in the mitochondrial respiratory chain and function as a cofactor for mitochondrial enzymes [40]. Human *COQ4 *is an interesting candidate gene for patients with CoQ_10 _deficiency or with developing isolated myopathy with progressive muscle weakness [41]. In pig, several QTLs were detected for fatness and growth at the position of the cluster [42-45]. The results of these different studies suggest that the cluster of eQTLs on chromosome 1 probably regulate cell death via mitochondrial respiratory function and subsequently muscle physiology. *COQ4 *could be an interesting candidate gene for further experiments to characterize alleles and how this allelic variation is affecting *COQ4 *gene expression and possibly muscle functions.

### Two putative *cis*-eQTLs are regulated by SSC5 and are tightly flanking the *HOXC *cluster

RNA levels of two genes, *PCBP2 *and *HNRNPA1*, were found to be genetically determined by two co-localized eQTLs, along with their own transcription unit (*cis*-eQTL), on the same segment of the distal arm of chromosome 5 (Figure 6A). These two eQTLs are part of a larger group of eQTLs at the same location (139-190 cM, but not identified as a cluster) where the two transcripts form a distinct transcription module as evidenced by the eQTL effects on these RNA levels (Figure 6B). It is striking that among the small number of *cis*-eQTLs found in our experiment, two were located in at neighboring positions and were co-regulated. This suggests shared dependence of the same *cis*-localized polymorphism through a transcription control mechanism acting locally. This is in contrast with the mechanism which is generally expected in clusters of *trans*-eQTLs, where a causative polymorphism may affect the downstream expression of a target gene and this gene, e.g. transcription factor, affect the expression of the other genes involved in the cluster.

The two genes tightly flank the *HOXC *cluster (*PCBP2 *is localized 200 kb 5' from *HOXC13*, *HNRPA1 *is localized 50 kb 3' from *HOXC5*, according to the genomic sequence version Sscrofa9). It has been reported that the *HOXA *and *HOXC *clusters are involved in the control of adult skeletal muscle differentiation [46] and although *HOXC *genes were not identified as eQTL co-segregating at this particular position, *HOX *genes are prototypes of multigenic *cis*-regulatory modules [47]. The *cis*-regulon observed in our study brackets the *HOXC *cluster, whereas evolutionary analysis [48] points to the consolidation of *HOX *clusters in vertebrates through selection of *cis*-regulons as better co-regulation systems. It is tempting to speculate that the flanking genes identified here were co-opted by a local *cis*-regulon including the *HOXC *cluster to drive an adult skeletal muscle differentiation program. All the more, as reviewed by Williams *et al*. (2007), *PCBP2 *and *HNRNPA1 *are plausible sites of action for genetic determinants of mRNA levels [29]. These two genes are known to be heterogeneous nuclear ribonucleoproteins (hnRNPs). HNRNPA1 is involved in pre-mRNA processing, polyA+ mRNA transport from nucleus to cytoplasm and alternative splicing control [48]. PCBP2 is a poly(C) binding protein known to control translation of specific mRNA through competitive binding of C-rich tracts in target genes 5'UTR and mir-328 [49]. PCBP2 has been shown to provide for selective expression of cell survival factors [50]. These functions may support control of a cell differentiation program linked to a stress response.

## Conclusion

Identifying the genetic regulation of gene expression is a new powerful way to decipher complex traits such as mammalian disease or livestock production traits. We can now describe phenotypic variations not only through genetic analysis, not only with differentially expressed genes but also with genetic analysis of gene expression of thousands of intermediate phenotypes. Integrated analyses across all these data types may help to more efficiently and more accurately identify causative polymorphisms or understand the molecular events involved in phenotype construction even when these are affected by several QTLs [21,51]. In this study, we identified 335 eQTLs of which half co-localized at six loci, suggesting co-regulation by the same polymorphims and subsequently, co-functions. The other half of the eQTLs provide information about the genetic control of the expression of specific genes. In addition, all the affected genes participate in a consistent set of biological functions, since when functionnal annotation is possible, functionnal networks can be contructed.

Beside the analysis of genetic variability in gene expression presented here, this systematic dataset will also offer opportunities in a priori analysis of expression levels correlations structure as for example inferring co-expression networks [52]. In future experiments, we expect to be able to combine trait-related gene expression and QTL analysis to propose positional candidate genes as underlying trait QTL and contribute to the identification of the causative polymorphisms.

## Methods

### Animals and Muscle samples

A group of 57 half-sib and full-sib pigs was selected from a larger F2 resource population, produced as an intercross between 16 F0 males and 25 F0 females from two production sire lines FH016 (Pietrain type, France Hybrides SA, St. Jean de Braye, France) and FH019 (Synthetic line from Duroc, Hampshire and Large White founders, France Hybrides SA, St. Jean de Braye, France), respectively. The two parental lines differed marginally in adiposity (backfat thickness) and longissimus muscle developpement (cross-section surface), although both were close to European pork production standards. The whole population included 1,370 F2 animals, progeny of 18 F1 males and 72 F1 females, and was used as a QTL detection resource population [53]. Animals considered in the present work were 33 females and 24 barrows selected from the largest half-sib family, representative of overall population variability in carcass and meat quality traits. These 57 animals where produced by three F1 sows mated with the same F1 boar for one to three litters, and were genotyped as non-carriers of either the RYR1 Cys^615 ^or the PRKAG3 Gln^200 ^allele, known to alter substantially the longissimus muscle physiology [54,55]. Muscle samples were biopsied from *Longissimus lumborum *(LD) muscle 20' after stunning and exsanguination. Samples were immediately frozen in liquid N_2 _and kept at -80°C until analysis. Procedures and facilities were approved by the French Veterinary Services.

### Genetic markers

Genomic DNA was extracted from piglet tails docked at birth, using a QIAGEN DNAg extraction kit, following manufacturer protocol. 170 microsatellites loci spanning the 18 autosomes with an average spacing of 17 cM were selected based on informativity on F1 animals and genotyped by PCR amplification with fluorescently labeled primers followed by denaturing acrylamide gel electrophoresis, revealed within a LICOR 4200 automated sequencing system. Allele calls were performed based on comigration with molecular weight standards using LICOR SAGA genotyping software. F2, F1 and F0 animals were all genotyped and Mendelian segregation was checked. Custom genetic maps were reconstructed with CRIMAP software [56]. The 170 microsatellites markers are described in Additional file 2 with position on genetic map and the corresponding genomic localizations. These localizations where estimated from genetic markers STS sequence homologies on genome sequence assembly using blat program (Ensembl Sscrofa9, see *in silico *Genomic localization).

### Total RNA extraction

The total RNA extraction was previously described [57]. Total RNA was isolated from each of the 57 muscle samples. Briefly, the muscle samples were disrupted, homogenized and ground to a fine powder by rapid agitation for 1 min in a liquid-nitrogen cooled grinder with stainless steel beads. An aliquot of 250-300 mg of the fine powder was then processed for total RNA isolation and purification using RNeasy Fibrous Tissue Midi kit according to the manufacturer's instructions (Qiagen SA France, Courtaboeuf, France). The method included a proteinase K digestion step to remove proteins and a DNase digestion step to remove contaminating DNA. The extracted total RNA was eluted in 300 μl of RNase-free water and stored at -80°C. RNA quality and concentration were controlled using an AGILENT 2100 bioanalyzer (RNA solutions and RNA 6000 Nano Lab- Chip Kit, Agilent Technologies France, Massy, France).

### Design and hybridization of cDNA arrays

The 9 K micro-array (GEO accession number GPL3729) used in this work was previously described [58]. The microarray Nylon cDNA hybridization and quantification using BZScan were the same as in [57]. The cDNA arrays were first hybridized with a vector oligonucleotide labeled with γ^33^P-ATP at 42°C for 12 h to determine the quality of the spotting process. After washing, the arrays were exposed for 6 or 24 h to radioisotopic sensitive imaging plates (BAS-2025, Fujifilm, Raytest France S.A.R.L., Courbevoie, France). The imaging plates were scanned thereafter with a phosphor imaging system at 25 μm resolution (BAS-5000, Fujifilm, Raytest France S.A.R.L., Courbevoie, France). The arrays were then stripped and hybridized with a complex target. Briefly, cDNA was synthesized and labeled from 5 μg total RNA by simultaneous reverse transcription of mRNA using SuperScript II RNase H-Reverse Transcriptase (Invitrogen SARL, Life Technologies, Cergy Pontoise) and α^33^P-deoxy-CTP. The mRNA of each muscle sample was hybridized at 68°C for 24 h to one array. Exposition and scanning were done as for vector hybridization. The hybridization images from vector and complex targets were quantified using the semi-automated software, BZScan [59]. Fixed circle segmentation, i.e. a grid process with a fixed spot diameter was applied. The grid obtained with vector hybridization images was used to assess the reproducibility of the quantification signals between array replicates and also to analyze the complex target hybridization images. The complex hybridization images were quantified by extracting the intensity of each spot. The microarray data from this research has been deposited in the NCBI Gene Expression Omnibus data repository under accession number GSE26924.

### Normalization/filtering of raw data

Data from BZScan outputs were normalized by the following process. First the vector oligonucleotides hybridization has been used to exclude spots without enough DNA spotted; this is in order to have an accurate hybridization with the muscle RNA sample. Then data were log base 10 transformed. 2,464 clones with signal intensity higher than 2 times the median value of background (water, plasmid and empty spots). Data were centered with the median value obtained from the arrays within each hybridization experiment. As we observed a non-linear median across samples, we used the loess function of R to adjust the medians for each sample. Finally, hybridization data have been centered for each spot. Normalization has been done using R software (R, language and environment for statistical computing, R Foundation for statistical computing, Vienna, Austria, http://www.cran.r-project.org).

### Genetic analysis

All normalized gene expression levels (2,464 spots selected) were first analysed for polygenic additive variation with an animal model using ASREML 2.0 software [60]. We estimated a narrow sense heritability (h^2^) for each e-trait as h^2 ^= VA/(VA+VE), where VA is the estimated additive genetic variance for the expression level of a transcript and VE is the estimated residual variance. All transcripts associated with an estimated heritability higher than 0.05 (1,057 transcripts) where then subjected to QTL analyse using a variance component approach [61]. Briefly, univariate mixed models of variance were fitted for each expression trait, using as fixed effects slaughter batch (7 levels), hybridization batch (2 levels) and spotting batch (2 levels). Additive genetic effect was fitted in an animal model, using a 3 generations pedigree structure to setup animal relationship matrix, and QTL effect was fitted using Identical By Descent (IBD) relationship matrix for the given genome position. IBD relationship matrices were estimated using package LOKI 2.4.6 [62], and variance components were estimated using Residual Maximum Likelihood (REML) method with ASREML 2.0 software [60]. A QTL detection test was computed each 2 cM along linkage groups using as a Likelihood Ratio Test (LRT), -2*(log(Additive and QTL model Likelihood)-log(Additive only model likelihood). We determined an empirical significance threshold within each linkage group from the distribution of LRT observed on simulated data under null hypothesis (no QTL). We simulated phenotypic data only, using same marker data and family structure and modeling only additive genetic variation within this pedigree structure (h^2 ^= 0.2). We generated 1,000 simulated datasets, which we further submitted to the same QTL detection procedure, recording distribution of maximum LRT values by chromosome (i.e. distribution of tests when no QTL contributes to variation) when scanning all genome positions. We then set 0.99 quantile of these LRT distributions for each chromosome, as a 1% chromosome-wise threshold for detection of eQTL.

While we detected 6 to 10 eQTL by chromosome with LRT higher that this threshold overall from 1,000 simulated data, when using the 1,057 heritable gene expression levels, we detected from 6 to 140 eQTL by chromosome, which suggests an achieved FDR for eQTL detection of 100% (no eQTL detected) to (2.4 × 10/140 = 17%), according to chromosomes. Those FDR estimates are likely very conservative, as many expression levels are highly correlated, and thus far to be modelled as 2,464 independent variables. All the results are given in Additional file 3 and summarized in Additional file 1.

However, as we have proposed a biological interpretation for all 335 eQTL identified as significant at a 1% chromosome-specific chromosome-wise threshold, including for each chromosome, the 10 false-positive eQTL that would be identified by chance for each chromosome if testing for 1,057 independent variables, we investigated realized FDR when considering all of the identified 335 eQTL, and taking into account the dependence among expression levels. We followed the approach suggested [63] and applied to this multiple trait and multiple chromosome testing issue [43] using a canonical transformation of traits based on phenotypic correlation structure, to estimate the number of independent variables being tested. The 1,057 expression traits having been subjected to the eQTL scan can be described with 55 independent eigenvectors, accounting for 99.6% of total variation (R software). Using a Bonferroni correction for testing of 18 independent chromosomes, a 1% chromosme-wise threshold applied to these 55 equivalent independent traits would give rise to 10 false positive eQTL overall. We identified 335 eQTL from 272 spots or RNA expression levels (1.23 eQTL/spot), accounting for a similar dimensionality of 54 independent traits in positive results. Positive results can be expressed as 67 (1.23 × 54) independent eQTL, thus our proposition of an overall FDR of 15% (10/67) when considering all of the 335 eQTL.

### Correlation structure of predicted eQTL effects and residuals

The correlation structure of predicted eQTL effects on expression levels for the transcripts associated with significant eQTL localized in the same genomic region was visually explored using correlograms as proposed [20] and implemented in R/corrgram software. The predicted eQTL effects were the solutions of the eQTL random effect in mixed model analysis at the selected genome position, as computed by ASREML 2.0 at model convergence. Residuals were collected from the same runs and subjected to the same correlation analysis among transcripts showing colocalized eQTL. The corrgram function produces a graphical display of a correlation matrix, called a correlogram. For each pair of expression measurements, color intensity and circle fill level are proportional to the absolute value of the corresponding correlation. The circles are filled clockwise for positive values, and anti-clockwise for negative values. The elliptic representations are depicting the patterns of relations among variables. The combination of concentration ellipses and loess smooth summaries of linear and possibly nonlinear association. The ellipse have their eccentricity parametrically scaled to the correlation value.

### *In silico *Genomic localization

To evaluate if the 272 genes could be associated with genomic region governing any eQTL region, we have identified their chromosomal localizations on the porcine genome. As the porcine genome is not completely sequenced [64], an *in silico *process was undertaken to ensure pig genome localization (Additional file 1). The same process has been applied for the 170 genetic markers (Additional file 2).

1) The pig ESTs were mapped to the pig genome (Sscrofa9.2) using the Narcisse alignment tool [65]. Only mapping location exhibiting more than 94% identity were considered as reliable (Ensembl http://www.ensembl.org/Multi/blastview, Sscrofa9, April 2009). 2) A systematic porcine localization was done against the High Throughput Genomic Sequences (HTGS, NCBI). The blast results (88%, 239/272) correspond to a BAC sequence previously localized on the porcine physical map and on the genomic human sequence by homology [66]. 3) When only a gene name is obtained from the cDNA annotation, the human localization of the corresponding gene are recovered and the results were compared to expected localizations from IMpRH web server [65] and the comparative human-pig map [67]. Comparative genomics were used for the 15 remaining transcripts with only a human gene annotation, but most transcripts were localized directely on porcine chromosomes. Fourteen transcripts have not been localized at the time of the analysis.

### eQTL distribution over genome and *cis*-eQTL detection

The observed distribution of eQTL most likely positions over the whole genome was compared to the expected distribution of these eQTL when assuming an equiprobable distribution of eQTL locations all over the 18 autosomes. A chisquare statistic was computed for the windows of 40 cM or more, where several eQTL co-localized. The six clusters of eQTL referred as such in results and in Table 1 were each associated to a highly significant chisquare test for enrichment of eQTL in the referenced genome segment (p-value < 10^-5^). These p-values have been adjusted with a Bonferroni correction. Details are given in Additional file 4.

The eQTL localizations are only broadly defined as a result of the long range linkage in a F2 population. We considered as putative *cis*-eQTLs those where genetic markers flanking the most likely eQTL position also bracketed the gene position at the 1% chromosome-wide significant eQTL detection on the genetic map. We also considered a more restrictive definition of *cis*-eQTL, including all eQTL where gene transcription unit was located within 25 Mb of the closest genetic marker. We qualified eQTL as putative *cis*-eQTL when coordinates of gene transcription unit on Sscrofa9 genome assembly were found within the interval defined by the genomic locations on the same assembly of the genetic markers bracketing the QTL closest marker position. We estimated the expected number of *cis*-eQTL as following a binomial law with a nominal probability of 1/(number of markers intervals/2), applied to 335 eQTL. Expected number of *cis*-eQTL detected by chance with this criteria would have a mean of 3.94 *cis*-eQTL, and a p-value lower than 0.01 of being higher than 9. It results in subselecting 15 *cis*-eQTL with an average distance of 7.8 Mb between the closest genetic marker and gene transcription unit. Both ways to identify the 18 putative *cis*-eQTL are given in the Additional file 3.

### cDNA clone annotation

The cDNA annotation was mostly done by homology with other complete genome such as the human or bovine genomes. The 272 transcripts were annotated searching sequence homologies against following databases: SwissProt, TIGR Pig SsGI 12, UniGene Pig, Ensembl Human Transcripts NCBI36 (annotation from SIGENAE, http://www.sigenae.org/). Finally, the 272 genes were manually annotated with blastn against the Refseq_RNA library (NCBI). The results are summarized in Additional file 1 and details are given in Additional file 5. E-values thresholds filtering alignments between Sigenae contigs and other databanks are: UniProt/RefSeq proteins, 10^-05^; RefSeq RNA, 10^-05^; UniGene/TIGR contigs, 10^-02^. For SIGENAE, hits are the most homologous sequences to our consensus sequences, after a BlastX (nucleotid/protein) on SWISSPROT. Only the best 20 first hits are recovered, with at least a score of 100 and an E-value of 10^-5^.

Manually, with NCBI/Refseq or Genbank databases, the observed thresholds are score 100, E-value 10^-16^, identity 74% (pig compared to another mammalian) or 91% (pig-pig comparisons). We verified the correspondence between the gene name and the genomic localization using comparative genomics between human and pig. Finally, a consensus gene name was kept.

### Functional annotation

The software EASE (the Expression Analysis Systematic Explorer; http://david.abcc.ncifcrf.gov/ease/ease.jsp) was used to obtain functional Gene Ontology (GO) terms for each gene. The systematic ontological annotation is given in Additional file 6. The Ingenuity Pathways Analysis (IPA, http://ingenuity.com/) application was used to identify the biological mechanisms, pathways and functions involving genes affected by eQTL. Statistically enrichment functions have been determined from the 145 eligible genes (Additional file 7) and illustrated with a pie chart (Figure 3). This system, a web-based interface, provides computational algorithms to identify and dynamically generate significant biological networks and pathways that are particularly enriched with our genes of interest. It also ranks networks by a score that takes into account the number of focus genes and the size of the networks, indicating the likelihood of the focus genes in a network being found together by chance. The higher the score (score = -log (p-value)), the lower is the probability of finding the observed Network Eligible Molecules in a given network by chance. We chose networks with direct relationships between genes. The detailed gene names presented in each network, cellular localization and function are described in Additional file 8. Main functions corresponding to each network are given by IPA after a statistical analysis of the significance of the included genes. The software tool Path Designer (IPA) was used to improve the readability of the networks.

## List of abbreviations

QTL: quantitative trait loci; eQTL expression quantitative trait loci; LRT: likelihood ratio test; SSC: Sus scrofa chromosome; GO: Gene Ontology; FDR: false discovery rate; IPA: Ingenuity Pathways Analysis; STS: Sequence Target Sequence

## Authors' contributions

LL, PLR and PC conceived and designed the study. NI, JG and PC designed the panel of genetic markers. JG, JP and PC collected samples and performed genotyping. VL, LL, AT and FB conducted the microarray experiments. MSC and LL analyzed the transcriptome data. PC conducted eQTL analysis. TF, AR and LL did the *in silico *genomic localization of the genetic markers and the genes. LL did gene annotation, gene networks, data interpretation and wrote the paper. PC and MSC contributed to data interpretation. PC and MSC helped draft the manuscript. All the authors read and approved the final manuscript.

## Supplementary Material

Additional file 1 **Description of the 335 eQTLs for 272 genes with heritability and genomic localization**. For each gene/accession number, this file gives the estimated heritability, the number of eQTL, the chromosome localization of the eQTL with the LRT value, the gene name, the gene localization on Sscrofa9 or on BAC chromosome or predicted localization by human-pig comparative genomic, and if the eQTL is a putative *cis*-eQTL (when associated with ** or ***) Click here for file

Additional file 2 **Description of the microsatellite markers used for the eQTL study**. Markers are positioned on genetic map (H.c6: Haldane distance units, cM), as estimated on resource F2 population and used in QTL detection procedures. This table also gives the putative genomic localization when possible. The last column gives the statistical threshold obtained after simulation. Click here for file

Additional file 3 **Description of all the 335 eQTL located all along the genome**. This file gives details for all the 335 LRT value (maximum for each chromosome) for each gene/accession number, the estimated heritability and the genetic localization (cM) of the eQTL. The genes involved in one of the six clusters of eQTL are in bold. The definition of the putative *cis*-eQTL is given according two ways: comparison of the interval of the genetic localization of the gene (cM) with the eQTL interval for putative *cis*-eQTL at 1% chromosome-wide significant eQTL detection (cM), and calcul of the position between the LRTmax to the genomic localization of the gene (Mb). Click here for file

Additional file 4 **Six significant clusters of *trans*-eQTL were identified**. The observed distribution of eQTL most likely positions over the whole genome was compared to the expected distribution of these eQTL when assuming an equiprobable distribution of eQTL locations all over the 18 autosomes. A chisquare statistic was computed for a window of 40 cM or more, where several eQTL co-localized. These p-values have been adjusted with a Bonferroni correction. Click here for file

Additional file 5 **Detailed annotation (gene and genomic localization) for the 272 genes**. For gene/transcript, the first annotation was obtained by the Sigenae bioinformatic platform. Next are given results from NCBI blast with (human genome and transcript) or transcript reference (Refseq) or Unigene or Ensembl databases. Transcript were mapped with Sigenae, with NCBI blast against HTGS database and Narcisse software for the genomic sequence or Blat with Ensembl genome browser (Sscrofa9). The consensus gene annotation and gene mapping are given in Additional file 1. Click here for file

Additional file 6 **The EASE web software provided funtional information for 127 of the annotated genes (from the 272 genes with at least one eQTL)**. For each annotated gene (when a gene symbol is available), this file gives the gene description, the human gene identifiers, the alias symbols, the Gene Ontologies (GO: Biological Process, Cellular Component, Molecular Function), the KEGG (Kyoto Encyclopedia of Genes and Genomes) pathway, and a summary of the functions of the gene when available. Click here for file

Additional file 7 **The 28 top biological functions identified by Ingenuity Pathways Analysis (p-value < 0.01)**. This table corresponds to Figure 3 and shows the co-ocurrence annotations found by IPA. Click here for file

Additional file 8 **Three significant gene networks were obtained with Ingenuity Pathways Analysis for the 186 annotated genes (out of the 272 genes) associated with eQTLs**. Detailed information about the genes included in each network are given in the neighboring columns: gene symbol, synonym(s), the Entrez Gene Name, in which networks the corresponding gene is involved, the subcellular location, the type(s) of the encoded protein, the Entrez Gene ID for Human. The three networks are presented with their description and the merged network. Genes in green are genetically regulated by a putative *cis*-eQTL. Genes in red are genetically regulated. Network 8.1: score 81, 57 genes/eQTL, cellular movement, cell-to-cell signaling and interaction, system development and function. Network 8.2: score 79, 55 genes/eQTL, post-translational modification, organ morphology, organismal injury and abnormalities. Network 8.3: score 61, 47 genes/eQTL, protein synthesis, drug metabolism, small molecule biochemistry. Network 5.4: A merged network between the three first networks with five genes shared by network 1 and 2, three genes shared by networks 1 and 3, and one gene shared by networks 2 and 3. Click here for file
